# Production of Porous β-Type Ti–40Nb Alloy for Biomedical Applications: Comparison of Selective Laser Melting and Hot Pressing

**DOI:** 10.3390/ma6125700

**Published:** 2013-12-06

**Authors:** Ksenia Zhuravleva, Matthias Bönisch, Konda Gokuldoss Prashanth, Ute Hempel, Arne Helth, Thomas Gemming, Mariana Calin, Sergio Scudino, Ludwig Schultz, Jürgen Eckert, Annett Gebert

**Affiliations:** 1 Leibniz Institute for Solid State and Materials Research IFW Dresden, P.O. Box 270016, Dresden D-01171, Germany; E-Mails: m.boenisch@ifw-dresden.de (M.B.); k.g.prashanth@ifw-dresden.de (K.G.P.); a.helth@ifw-dresden.de (A.H.); t.gemming@ifw-dresden.de (T.G.); m.calin@ifw-dresden.de (M.C.); s.scudino@ifw-dresden.de (S.S.); l.schultz@ifw-dresden.de (L.S.); j.eckert@ifw-dresden.de (J.E.); a.gebert@ifw-dresden.de (A.G.); 2 Institute of Materials Science, TU Dresden, Helmholtzstr. 7, Dresden D-01062, Germany; 3 Institute of Physiological Chemistry, Carl Gustav Carus Faculty of Medicine, TU Dresden, Fiedlerstr. 42, Dresden D-01307, Germany; E-Mail: hempel-u@mail.zih.tu-dresden.de

**Keywords:** novel β-phase Ti-based alloys, static biomechanical behavior, cytotoxicity and cell proliferation

## Abstract

We used selective laser melting (SLM) and hot pressing of mechanically-alloyed β-type Ti–40Nb powder to fabricate macroporous bulk specimens (solid cylinders). The total porosity, compressive strength, and compressive elastic modulus of the SLM-fabricated material were determined as 17% ± 1%, 968 ± 8 MPa, and 33 ± 2 GPa, respectively. The alloy’s elastic modulus is comparable to that of healthy cancellous bone. The comparable results for the hot-pressed material were 3% ± 2%, 1400 ± 19 MPa, and 77 ± 3 GPa. This difference in mechanical properties results from different porosity and phase composition of the two alloys. Both SLM-fabricated and hot-pressed cylinders demonstrated good *in vitro* biocompatibility. The presented results suggest that the SLM-fabricated alloy may be preferable to the hot-pressed alloy for biomedical applications, such as the manufacture of load-bearing metallic components for total joint replacements.

## 1. Introduction

Titanium (Ti) and its alloys are widely used as load-bearing implant materials for hard tissue support and replacement because of good mechanical properties, excellent biocompatibility, and high corrosion resistance [1]. One of the shortcomings of commonly-used Ti-based alloys is high stiffness, expressed as high modulus of elasticity (*E*) (typically, >100 GPa). In implant applications, a large stiffness mismatch between the implant material and the contiguous bone can lead to stress shielding, which retards the mechanical stimulation of the bone healing process [2]. Thus, for a given Ti-based alloy, it is desirable to reduce its *E* to that of healthy bone (4–30 GPa) while maintaining its high strength and good plasticity [3]. Two approaches have been taken to achieve this goal. One is the production of metastable β-type wrought titanium-niobium (Ti–Nb) alloys (for example, Ti–40Nb), but the reported minimum value of *E* (60–62 GPa) is too high [4]. Alternatively, porous Ti–Nb alloys, having a microstructure similar to that of cancellous bone, have been produced [5].

There are a fair number of literature reports on production and characterization of porous Ti–Nb alloy. Lin *et al*. [6] used powder, produced using ball-milled powder and a powder metallurgical method to fabricate Ti–35Nb porous samples. The samples were produced by sintering with ammonium bicarbonate particles as space-holder. Yang *et al*. [7] used powder produced by high energy vibration ball milling and gel casting to fabricate Ti–25Nb samples. Fojt *et al*. [8] used commercial titanium and niobium powders and PM to fabricate Ti–39Nb. Zhuravleva *et al*. [9] used ball-milled Ti–40Nb powder and a space-holder method to fabricate porous samples. One of the methods that has recently been introduced for the production of porous alloys is selective laser melting (SLM). The process involves direct melting of a powder and creation of net-shaped bodies through a “layer by layer” approach. Each layer is melted by a scanning laser and is mounted on a previously molten layer. The high temperature, steep temperature gradient, and fast cooling rates involved in SLM allow stabilization of metastable phases in an alloy [10]. To the best of our knowledge there are neither studies involving the use of SLM to fabricate porous Ti–Nb alloys nor studies involving comparison of properties of a Ti–Nb alloy fabricated using different techniques but with the powder produced using the same method.

The purpose of the present work was to compare the properties/characteristics of Ti–40Nb alloy fabricated using SLM and employing a better established method (hot pressing), with the powder produced using the same method (mechanical alloying) in both cases. The properties/characteristics determined were phase composition, morphology, total porosity, inner pore architecture, compressive strength, compressive modulus, and *in vitro* biocompatibility.

## 2. Experimental Section

The starting materials were Ti powder from TLS Technik, Bitterfeld, Germany (purity 99.2%, <100 mesh, spherical shape) and Nb powder from Johnson Matthey GmbH, Karlsruhe, Germany (purity 99.8%, 325 mesh, irregular shape). The Ti–40Nb alloy powder was synthesized using mechanical alloying (MA), a process in which the elemental starting powders and a lubricant are repeatedly cold-welded and fractured under the action of high-energy collisions of the balls in a container (ball milling operation). In the present work, the anti-sticking agent (lubricant) used was 2 wt % NaCl [11].

The ball milling was carried out in a Retsch planetary ball mill under argon atmosphere with vials and balls made of C15 steel. The rotation velocity was 250 rpm and the milling duration was 40 h with pauses of 15 min every 15 min to let the vials cool down. These milling parameters were found to result in the lowest oxygen content in the final powders. The ball-milled powder was sieved before SLM processing to limit the particle dimensions to 20–44 µm. The flowability of the powder was tested by allowing the powder to flow through an orifice and was evaluated as very good. The phase composition of the milled powder was analyzed using a Bruker X-ray diffractometer (XRD) with CoKα radiation and its morphology was studied using a Jeol 6400 scanning electron microscope (SEM) (Jeol, München, Germany) equipped with a Noran EDX detector (Thermo Scientific, München, Germany).

Using the MA-produced alloy powder, solid cylindrical specimens with a diameter of 3 mm and a height of 6 mm were created with a 3D-CAD program (using Magics and MTT software) and were then built with an SLM 250 device by SLM solutions. The machine is equipped with oxygen sensors to enable low oxygen contents during the SLM process. As Ti and its alloys are reactive to oxygen, the chamber was filled with argon gas with a purity of 100 ppm before the process started. The following SLM process parameters were used: energy input: ~285 J/mm^3^; laser power; ~200 W; laser speed: 35 µm/s; layer thickness was 100 µm; specimen hatching distance: 200 µm; and hatch rotation: 74°. For the hot-pressed study group, the MA-produced powder was pressed at 600 °C and 500 MPa for 30 min in an argon atmosphere and then cooled inside the press chamber. Test specimens (solid cylinders, with diameter and height = 3 mm and 6 mm, respectively) were cut from the hot-pressed stock.

The phase composition of the cylinders was studied by XRD and morphology was determined using SEM and transmission electron microscopy Teccnai (TEM) (Fei, Gräfelfing, Germany). The TEM specimens were prepared by a focused ion beam (FIB) technique using a Gemini 1540 XB cross-beam machine at 30-keV Ga ion energy. To protect the lamella against the ion beam, a Pt layer was deposited at the front of the specimen. To keep the thin specimen stable, thick support bars were left in between very thin windows. The specimen was thatched to a 3 mm-thick M-shaped specimen holder and the thinning was stopped when holes appeared in the lamella.

The total porosity (pT) of the cylinders was determined by the Archimedes method with a Sartorius MC210P balance. The pore geometry was studied using SEM (JEOL 6400). The inner architecture of the specimen was analyzed with micro-CT by using a computed tomograph (Nanotom S; GE, Ahrensburg, Germany). The specimens were scanned at 120 kV with a voxel size of 4 mm. The compressive strength and the compressive modulus of elasticity (*E*) of the SLM-produced alloy were determined from compression tests carried out on the cylinders, at room temperature, at a strain rate of 10^−3^·s^−1^.

*In vitro* biocompatibility experiments were performed on specimens from all three study groups using human bone marrow stromal cells (hBMSC). For isolation of hBMSC, bone marrow aspirates were collected from bone marrow donors (age: 32 ± 3 years) at the Dresden Bone Marrow Transplantation Centre of the University Hospital Carl Gustav Carus. The study was approved by the local ethics commission (approval No. EK251072013). The donors were informed and gave their approval. hBMSC were isolated using the method described by Ostwald *et al*. [12]. Briefly, ~10 mL of bone marrow aspirate was diluted 1:5 with 0.5% human serum albumin (HSA) (Braun, Melsungen, Germany) in phosphate-buffered saline (PBS) (Biochrom, Berlin, Germany) and applied to a Percoll (Biochrom, Berlin, Germany) density gradient (*d* = 1.073 g/mL). After centrifugation at 900 × g for 30 min at 25 °C, mononuclear cells in the interface were harvested and filtered through a nylon cell strainer (100 µm, Becton Dickinson, Heidelberg, Germany). The cells were re-suspended in DMEM (Biochrom, Berlin, Germany) containing 10% heat-inactivated fetal calf serum FCS (Th. Geyer, Renningen, Germany) and antibiotics (Biochrom, Berlin, Germany). After 24 h, non-adherent cells were removed. When the adherent cells reached about 90% confluence, they were trypsinized with 0.05% trypsin/0.02% EDTA (v/v) (Biochrom, Berlin, Germany) in PBS and sub-cultured. For the experiments, 5000 hMSC/cm^2^ were deposited on the test specimen. Metabolic activity was determined by the MTS assay (Cell Titer96 AQueous One Solution Proliferation Assay) (Promega, Mannheim, Germany) 24 h after plating. Conditioned medium was replaced by fresh medium containing 10% of MTS dye solution. After 2 h of incubation at 37 °C in a humidified CO_2_ incubator, 80 mL cellular medium was transferred to a 96-well plate and the absorbance of the formed MTS formazan dye was measured photometrically at 490 or 655 nm.

In terms of statistics, the results of the quantitative parameters are presented as mean ± standard deviation (together with median and variance, in some cases). Significance of difference of the pT, ultimate compression strength, and *E* results between the two study groups was performed using the Mann-Whitney test, with significance denoted at *p* < 0.05.

## 3. Results and Discussion

### 3.1. Structural and Morphological Characterization of Powder and Alloy Specimens

The MA-produced powder particles have an irregular shape, with diameter of 24 ± 20 µm and consist of single β-(Ti,Nb) phase. The broadening of the peaks in the XRD spectra at different stages of the ball-milling process (not shown here) suggests the formation of an ultrafine grain structure and an accumulation of strain in the lattice, which is typical for ball-milling related to severe mechanical deformation [11]. The ball-milling process, the morphology and phase composition of the powder were discussed in detail in the previous work of the authors [13]. Ball-milled powder was sieved to select particle sizes of 20–45 µm and its flowability was determined to be very good. Therefore, the MA-produced powder was found to be suitable for use in both SLM and hot pressing.

**Figure 1:**
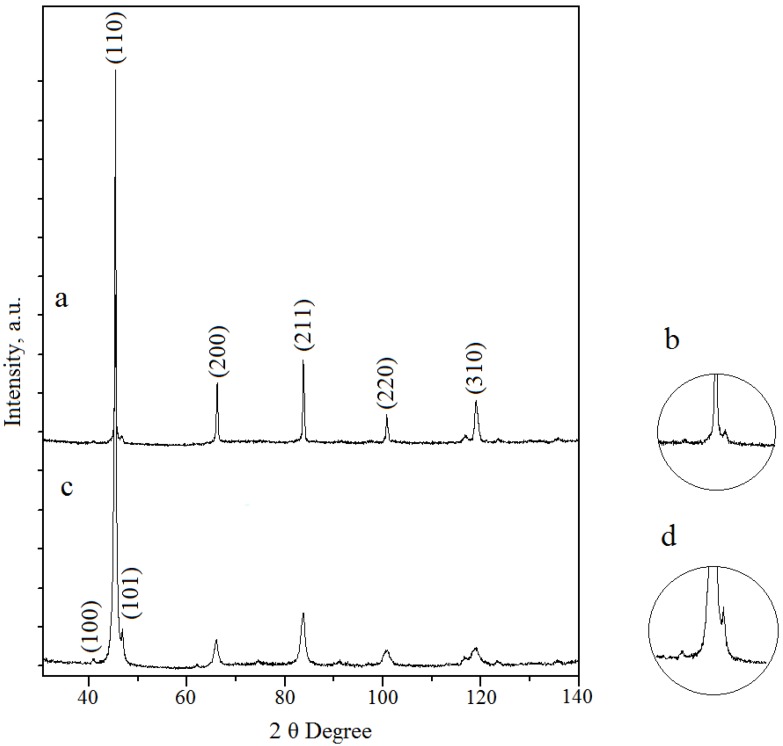
X-ray diffractometer (XRD) patterns of (**a**) sample made by selective laser melting (SLM) of Ti–40Nb ball-milled powder and (**b**) a higher resolution of its (101) peak; (**c**) sample made by hot-pressing of Ti–40Nb ball-milled powder and (**d**) a higher resolution of its (101) peak.

The XRD pattern taken from the cross-section of an SLM-fabricated cylinder is shown in Figure 1a,b. Phase analysis reveals that the sample consists mainly of β-phase and a small amount of a minor phase, which might be either α or α′′-phase. Martensitic α′′-phase fractions (orthorhombic structure, space group *Cmcm*) have been identified in some studies in Ti–Nb alloys which were rapidly quenched from the β-phase region [14]. The presence of an α′′-phase was also suggested by TEM studies and may be attributed to oxygen impurities in the ball-milled powder together with the limited cooling provided during SLM, which triggers its precipitation.

The XRD pattern taken from the cross-section of a hot-pressed cylinder is shown in Figure 1c,d. Both a main β-phase and a minor α-phase are identifiable. The amount of α-phase was evaluated as 11% ± 5% by the Le Bail method [15]. The appearance of α-phase can be mainly attributed to a slow cooling rate as the samples had to be cooled inside of the hot press chamber. After slow cooling, the presence of the ω phase precipitates (hexagonal structure, space group *P*6/*mmm*) is also possible because, according to the literature data ω-phase precipitates may form during a slow quenching from the β-phase region or during isothermal aging [14]. The presence of the ω-phase is hard to detect from the XRD patterns but it was confirmed by TEM studies in a previous work [13].

**Figure 2:**
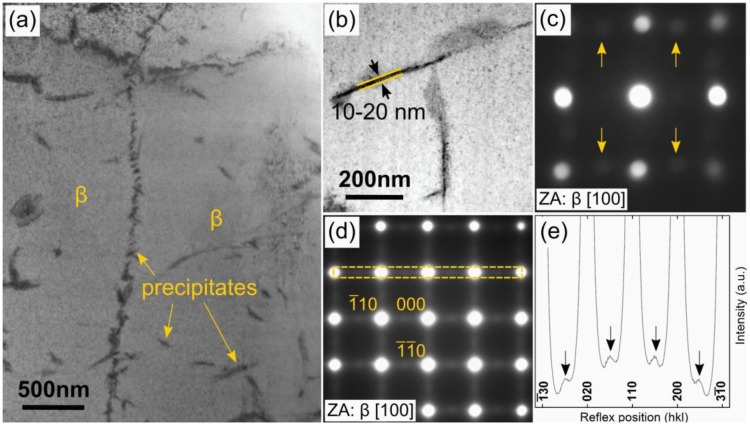
(**a**) Bright field transmission electron microscopy (BF-TEM) image of sample made by SLM of Ti–40Nb ball-milled powder showing the precipitates embedded in the β matrix. The orientation of β to the left and to the right of the vertical line of precipitates is identical; (**b**) Close up of a group of precipitates, some of them viewed edge-on. Their thickness varied between 10 and 20 nm; (**c**) Nano beam diffraction pattern recorded in [100]_β_ zone axis with the beam positioned on a single precipitate. The arrows mark reflexes not attributable to β; (**d**) Selected area electron diffraction (SAED)-pattern of the β matrix demonstrating the diffuse streaking along <110>_β_ reciprocal lattice directions; (**e**) Intensity profile of the rectangular area marked in (b) illustrating the intensity maxima centered between β reflexes along <110>_β_ reciprocal lattice directions.

TEM examination of selected regions of an SLM cylinder revealed precipitates with plate-like morphology embedded inside the metastable β-phase matrix (Figure 2). The bright-field (BF) TEM image (Figure 2a) shows a region with an area of about 9 µm^2^ vertically intersected by precipitates arranged along a line. Additional single precipitates can be found uniformly distributed on both sides of this line. The long dimension of the precipitates ranged from 50 to 450 nm whereas the thickness was 10–20 nm (Figure 2b) which represents a typical group of precipitates with a few of them oriented edge-on. Electron diffraction patterns using an electron beam with diameter of *ca.* 40 nm centered on one of the precipitates with the β-phase matrix oriented in [100] zone axis, exhibited additional weak reflexes (Figure 2c). Judging by their positions, they may be caused by either diffusional α- or by martensitic α′′-phase in [$\bar{1}\bar{1}0$] or [100] orientation, respectively. However, due to the combination of high cooling rates that occur during the SLM process, in conjunction with sluggish diffusional kinetics of the Ti–Nb system [14], it is postulated that the second phase particles are of martensitic α′′-type.

The β-phase matrix on both sides of the line of precipitates is oriented identically, no misorientation could be detected by recording selected area electron diffraction (SAED) patterns from both areas (Figure 2d). During SLM, small-scale pools are created by the laser, which scans across the powder. Consequently, part of the already consolidated material below the powder layer is also melted. This can result in epitaxial growth of grains across the SLM layers in the build direction [16]. However, it may be assumed that the atoms in the solidified material are not arranged perfectly but various types of structural defects on the atomic scale will be created due to high cooling rates and strong temperature gradients. This will be especially true for the outermost layers of atoms inside a single melt pool, which are closest to the liquid-solid interface between the melt and the substrate where solidification occurs first. Therefore, defect-rich layers will form at the melt-pool boundaries and will be present in the solidified material. The defects thus trapped will act as nucleation centers for the formation of secondary-phase particles during further cooling, thereby giving rise to non-homogeneous precipitate arrangements such as observed in the present work arrangements (Figure 2a).

Diffuse reciprocal lattice streakings are commonly observed in β-stabilized Ti alloys, the origin of which lies in structural instabilities inherent to the β-type *bcc* lattice. An SAED pattern corresponding to a circular area with diameter of 770 nm of the β-phase matrix oriented along [100]_β_ is presented in Figure 2d. In addition to the strong principal β-reflections, streaks of diffuse intensity were observed along <110> directions of the β reciprocal lattice. The intensity profile in Figure 2e of the rectangular region marked in the SAED-pattern (Figure 2b) clearly demonstrates that the diffuse intensities along the <110>_β_ reciprocal lattice directions form maxima at ½ positions between the principal β-phase Bragg reflections. Tahara *et al*. [17] and Nii *et al*. [18] reported similar streaking effects in the metastable β-phase matrix of Ti–Nb alloys with small oxygen (O) additions of about0.3 wt % (*ca.* 1 at %) These workers [17,18] concluded that the diffuse intensities arise from transversal {110}_β_<110>_β_ lattice modulations caused by local stress fields around O atoms randomly distributed on octahedral interstices in the bcc host lattice. Atom shuffles of the same type are necessary to form the martensitic α′′ structure (orthorhombic, *Cmcm*) from the parent β *bcc* lattice. The O level of the initial powder used for this study (0.38 wt %) was similar to levels in the alloys studied by Tahara *et al.* [17]. Based on their results [17], it is suggested that the diffuse intensities along <110>_β_ directions in reciprocal space observed in the present work are caused by {110}_β_<110>_β_ atom shuffles caused by strain fields around interstitial oxygen atoms.

**Figure 3:**
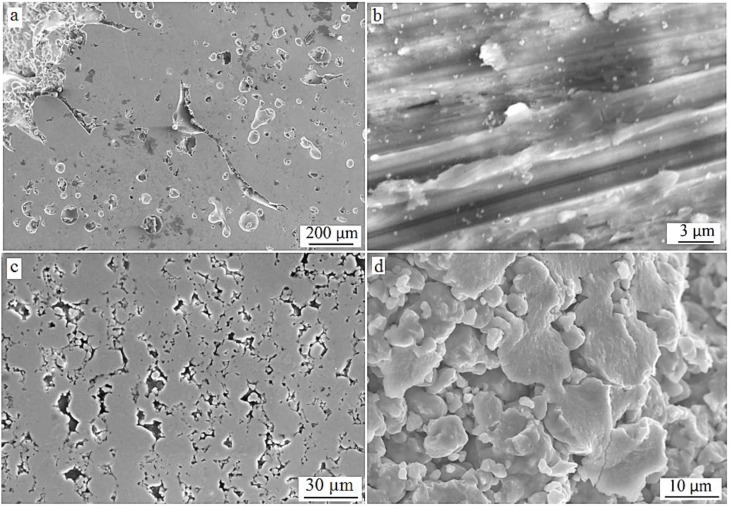
Scanning electron microscope (SEM) images of cross-sections of (**a**) sample made by SLM of Ti–40Nb ball-milled powder and (**b**) its higher resolution image; (**c**) sample made by hot-pressing of Ti–40Nb ball-milled powder and (**d**) its higher resolution image.

The microstructure of a typical cross-section of an SLM-fabricated cylinder is presented in Figure 3. The width of individual metal layers in these samples was found to be around ≈100 µm. There are two types of pores detectable in the image in Figure 3a: the elongated pores with a thickness of around 10 µm (formed by the incomplete connection between the molten layers) and small spherical-shaped pores of dimensions of 20–200 µm. The elongated pores are the consequence of the layer-by-layer approach in the SLM process and are typical for SLM fabricated alloys [16]. These pores may result from the scan track instabilities or sphereodisation of the liquid melt pool. The spherical pores have a relatively large size and form a high porosity level of the alloy. The reason for the formation of these spherical pores can be that the mechanical alloying yields irregular alloy particles with higher contamination levels of O, C, N [11]. Therefore, the particles have a more pronounced oxide layer on their surface than gas-atomized alloy particles. This can act as a thermal barrier for the local melting process during SLM and, thus, leads to formation of a high-porosity level.

In the hot-pressed cylinders , the pores were small and of irregular shapes since they were formed by the gaps between sintered powder particles [12] (Figure 3c,d). The powder particles are interconnected with each other via metal necks formed during the sintering. During hot pressing, the pressure from the punch was transported from one particle to another, deforming them and compacting the powder. The deformation in the contact points of the powder particles is elastic but, with growing pressure, causes plastic yield of the metal extending the contact points to contact areas [19].

The porosity (pT) for the SLM-fabricated cylinders was 17% ± 1% (median = 17.0%; variance: 0.4%), which is in good agreement with the value obtained using density determined by simply dividing the mass of the cylinder by its volume. As seen in Figure 3a, the porosity is mainly formed by spherical pores, which result from the gas uptake by the powder during the ball milling process. These big spherical pores and such a relatively high porosity are not typical for alloys fabricated using SLM from gas-atomized powders. The relative density of Ti alloys fabricated using gas-atomized powder and SLM is of the order of 99% [12,20,21]. For hot-pressed cylinders, the size of the micropores and pT were 3–12 µm and 3% ± 0.4% (median = 3.0%; variance = 0.06%), respectively. pT for SLM-fabricated cylinders was significantly higher than that for hot-pressed cylinders (*p* = 0.01).

**Figure 4:**
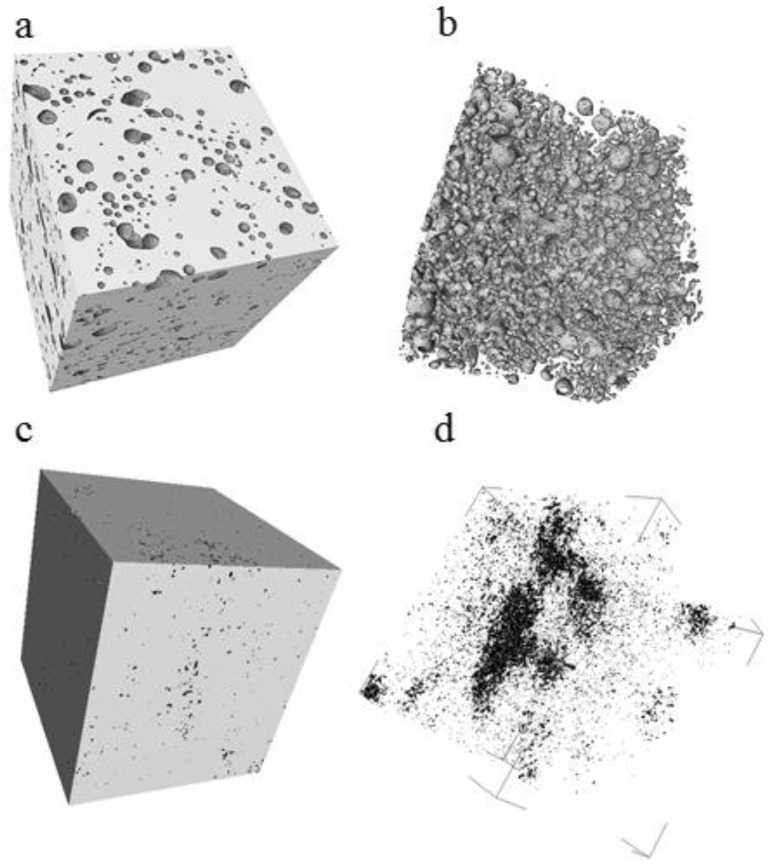
Micro-computed tomography (µCT) images of (**a**) sample made by SLM of Ti–40Nb ball-milled powder and (**b**) its inner porous architecture (from image analysis); (**c**) sample made by hot-pressing of Ti–40Nb ball-milled powder and (**d**) its inner porous architecture (from image analysis).

The reconstructed 3D image for an SLM-fabricated cylinder is shown in Figure 4a. The pores are perfectly spherical, consistent with the SEM images and are clearly visible in Figure 4b, which represents only pores (air) and no solid metal phase. The pores are homogeneously distributed all over the sample volume and are not interconnected. For the hot-pressed cylinders, the µCT studies of the hot-pressed sample (Figure 4c,d) revealed that the porosity is homogeneously destributed all over the volume of the sample. The resolution of the µCT was 4 µm, so it is possible that not all the porosity is visible on the images. The porosity is not interconnected and the shape of the pores was found to be irregular, consistent with the results given in Figure 3d.

### 3.2. Mechanical Testing

**Figure 5:**
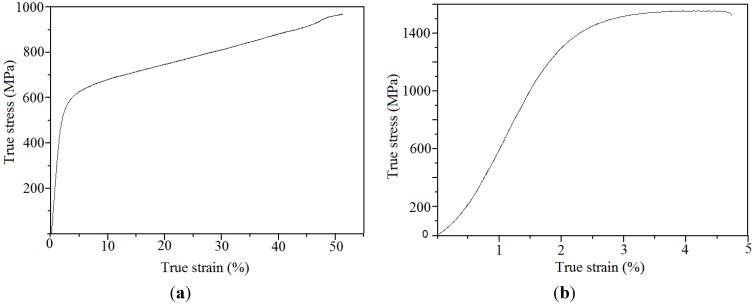
Stress-strain curves of (**a**) sample made by SLM of Ti–40Nb ball-milled powder and (**b**) sample made by hot-pressing of Ti–40Nb ball-milled powder.

Typical stress-strain curves are presented in Figure 5. UCS of the SLM-fabricated material (968 ± 8 MPa) (median = 964.5 MPa; variance = 58.4 MPa) was significantly lower than that for the hot-pressed material (1400 ± 19 MPa) (median = 1400 MPa; variance = 351 MPa) (*p* = 0.01). Two reasons are postulated to account for this difference. One is to do with the difference in bonding between the alloy powder particles at the end of the two fabrication processes. The temperatures during the SLM process are so high that the metallic powder particles are almost completely molten, because this process involves full melting of the powder [22]. This is a liquid state process that results in homogeneous alloy regions where no single powder particles are present (Figure 3b). The alloy powder particles in a hot-pressed sample are sintered together by forming small necks as shown in (Figure 3d). This is a consequence of the relatively low temperature of the hot-pressing process, *i.e.*, only 600 °C. This temperature is not high enough to completely melt the alloy powder particles. Besides that, the process time of 30 min eliminated grain growth and stress relaxation in the powder particles was eliminated during the process time (30 min) [23].

The second reason for the significant difference in the strength between SLM processed and hot-pressed samples is the porosity. Porosity has a strong influence on the mechanical properties and strongly reduces the strength of an alloy [5].

The Young’s modulus SLM-fabricated material (33 ± 2 GPa, median = 33.0 GPa; variance = 4.1 GPa) was significantly higher than that for the hot-pressed material (77.0 ± 3.1 GPa) (median 76.5 GPa; variance = 5.1 GPa) (*p* = 0.01). Two reasons are postulated to account for this difference.

First, the porosity of the hot-pressed material is very low so it cannot strongly reduce the stiffness of the alloy; Second, slow cooling of the cylinders inside of the press chamber lead to the formation of ω-phase, which has the highest elastic modulus among all the phases in the Ti–Nb system [14].

### 3.3. *In Vitro* Biocompatibility Performance

**Figure 6:**
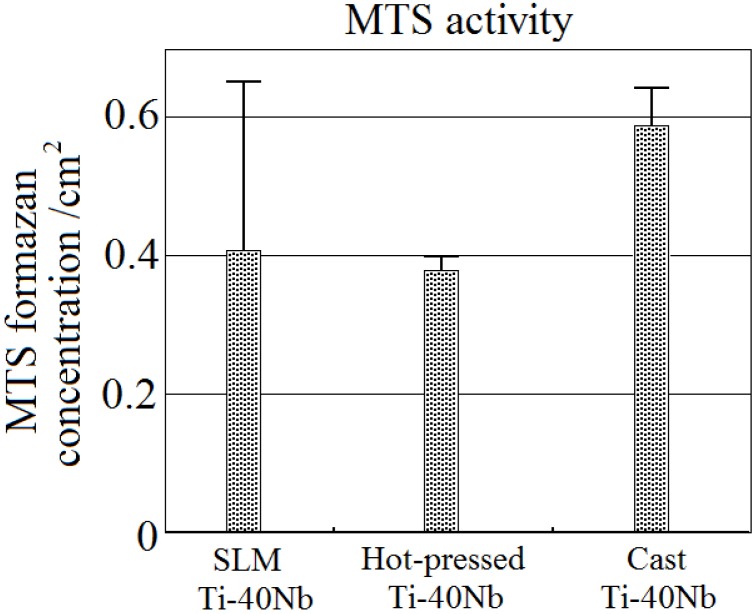
Metabolic activity of human bone marrow stromal cells (hBMSC) after 24 h of culture on a sample made by SLM of Ti–40Nb ball-milled powder, a sample made by hot-pressing of Ti–40Nb ball-milled powder and a cast Ti–40Nb sample determined by MTS assay.

The median MTS formazan concentration on hot-pressed cylinders was lower than that on SLM-fabricated cylinders (Figure 6), suggesting a better biocompatibility of the latter material. Cast β-type Ti–40Nb alloy samples were used as reference. hBMSC were analyzed for metabolic activity by MTS assay after 24 h (Figure 6). The surfaces of the reference cast samples was ground and polished with SiC emery paper down to P4000. The SLM Ti–40Nb samples were submitted to the cell tests as-processed. A typical surface of SLM samples has a layered structure and a rough topography, which is beneficial for cell attachment [24]. As first interpretation, less MTS formazan formation appeared to be observed on hot-pressed Ti–40Nb surfaces, but the difference between cast Ti–40Nb and SLM produced Ti–40Nb was not significant. However, it is unclear if the difference is significant because of the large scatter in the results in the case of SLM-fabricated cylinders, which may be attributed to their high surface roughness. Further cell tests are in progress to analyze these effects in more detail. Other key mechanical properties that are important for biomedical applications of alloys are fatigue life and fatigue crack propagation resistance. These properties of the alloys will be reported in further publications.

## 4. Conclusions

Solid cylinders were fabricated using β-type mechanically-alloyed Ti–40Nb powder and either SLM or hot pressing. These cylinders were compared on the basis of phase composition, morphology, porosity, inner pore architecture, compressive strength and modulus, and *in vitro* biocompatibility. The principal findings are as follows:

- In the SLM-fabricated material, there was a small amount of α or α′′ as a second phase in the β matrix, whereas hot-pressed material comprised (α + β) mixture with ω phase precipitation.
- In the SLM-fabricated material, porosity was formed by contamination of the powder with O, C, and N during the mechanical alloying, resulting in total porosity (pT) of 17% ± 1%, whereas in the hot-pressed material, porosity was formed between the powder particles, resulting in pT of 3% ± 0.4%. In both sets of cylinders, the porosity was not interconnected but was homogeneously distributed over the cylinder volume.
- Compressive strength and modulus of the SLM-fabricated material were 968 ± 8 MPa and 33 ± 2 GPa, respectively, whereas the corresponding values for the hot-pressed material were 968 ± 8 MPa and 33 ± 2 GPa. For each of these properties, the difference in results for the two materials may be attributed to the difference in phase composition and pT.
- Both SLM-fabricated and hot-pressed cylinders showed good biocompatibility.

These findings suggest that the combination of mechanically-alloyed Ti–40Nb powder and selective laser melting may have promise for use in manufacturing load-bearing metallic orthopaedic components, such as the femoral stem of a hip implant.
